# Editorial: Environmental extremes threatening food crops

**DOI:** 10.3389/fpls.2023.1172539

**Published:** 2023-04-14

**Authors:** Nasim Ahmad Yasin, Tanveer Alam Khan, Aamir Ali, Mukhtar Ahmed, Rehana Sardar

**Affiliations:** ^1^ RO-II Office, University of the Punjab, Lahore, Pakistan; ^2^ Botany, Leibniz Institute of Plant Genetics and Crop Plant Research (IPK), Gatersleben, Germany; ^3^ Botany, University of Sargodha, Sargodha, Pakistan; ^4^ Horticulture, Pir Mehr Ali Shah Arid Agriculture University, Rawalpindi, Pakistan; ^5^ Institute of Botany, University of the Punjab, Lahore, Pakistan

**Keywords:** abiotic stress, environmental factor, plant stress, stress alleviation, stress mitigation, tolerance

Food products are either directly obtained from the plants or indirectly associated with the food crops on a few steps in the food web. Life seems impossible without the unremitting supply of agricultural food products. However, cultivation and production of agronomic and horticultural food crops is highly dependent on the prevailing environmental conditions. Unsuitable environmental conditions including heavy metals contamination, drought, salinity, temperature extremes induce abiotic stresses which reduce growth, development and yield of the food crops. The extreme environmental stresses may cause a complete failure of the food crops which may lead to famine and starvation. Hence, it becomes necessary to evaluate the potential of various techniques to alleviate abiotic stresses in crop plants. Consequently, the Guest Editors of the Special Issue made a call for research articles *via* Frontiers in Plant Science, encouraging researchers to submit their research work on the issue, concentrating on crucial aspects which may help in solving associated environmental problems, with pertinence today and/or in the upcoming era. It is assumed that the outcome has been evidently productive. Manuscripts for this Special Issue come from Poland, China, United States, Canada, India, Italy and Pakistan. Researchers used various physicochemical, physicochemical and molecular techniques or resistant plant species to demonstrate the strategies for alleviation of plant stress. Total 54 articles were received, with final publication of 36 articles having the better quality, accepted after peer-reviewing.

There are 6 review articles and 30 original research articles. In his review article, Chen et al. made a review on the potential of secondary metabolites in alleviation of environmental stresses in crop plants. In the review article Ahmad et al. confers the contribution of black colored flowers in stress alleviation. Similarly, the review article of Ahmad et al. informs about the potential of plant growth promoting rhizobacteria in the alleviation of drought stress in crop plants. Farooq et al. have conferred the undesirable influences of climate change on cultivation of food crops. While, in their review article, Javed et al. have focused on the current developments in agronomic and physio-molecular tactics for improving nitrogen use efficiency in crop plants in their review article.

Environmental factors affecting plants growth is therefore the main topic of this special issue, subsequently confirming the emphasis of the researchers on the adverse effects of these factors either alone or in combination with other factors. Nickel stress decreases growth of pepper plants by affecting the activity of antioxidative enzymes, nutrients uptake, membranous integrity and biosynthesis of secondary metabolites (Altaf et al.). Temperature extremes may decrease the growth of *Brassica juncea* by affecting antioxidant system, synthesis of amino acids and soluble sugars (Chauhan et al.). The low temperature during post-silking stage of wheat reduces the enzymatic activity leading to the reduced biosynthesis of phytohormones, proteins and starch (Guo et al.). Plants subjected to water deficit conditions exhibit reduced stomatal conductivity, photosynthetic activity, water use efficiency, photochemical extinction, net CO_2_ assimilation, rate of transpiration, electron transport rate, carboxylation efficacy and PS-II activity (Javaid et al.). Temperature stress affects rice quality attributes such as chalkiness, protein content, pasting temperature, and starch content (Tu et al.). Climatic factors significantly modify phenology and grain quality of wheat (Poggi et al.). Waterlogged conditions modify protein biosynthesis, decrease synthesis of photosynthetic pigments as well as other metabolic enzymes, enhance photorespiration in wheat crop (Yang et al.).

On the other hand, abscisic acid and proline concentration have a decisive role in alleviation of salt and drought stress in *Salsola imbricata* (Alam et al.). The plasticity of the root system architecture modifies root volume, root surface area, root diameter and root length in *Lagenaria siceraria* to tolerate water-deficit situations (Contreras-Soto et al.). Estrada et al. studied the contribution of growth attributes including gas exchange parameters, chlorophyll fluorescence and spectral reflectance of different wheat genotypes in temperature and drought stress tolerance. According to Farooq et al., variations in hydraulics and xylem anatomy affect abiotic stress tolerance capability of different plant species.

Ahmad and Liu (2022) identified the possible involvement of myeloblastosis genes in cadmium stress tolerance of *Ipomoea aquatica* plants. It was observed that changes in RNA editing correspondingly alter amino acid synthesis in plants subjected to alkaline stress to tolerate adverse conditions (Rehman et al.). Sabir et al. studied the involvement of glutathione S-transferase gene family of *Prunus avium* in bioaccumulation of anthocyanin. Wang et al. found that GRAS genes may induce leaf formation and enhance abiotic stress tolerance in *Pyrus bretschneideri*. Plants capable of adjusting the expression level and binding interaction of *Superoxide Dismutase (SOD)* genes with hydrogen peroxide (H_2_O_2_) may tolerate the adverse environmental conditions (Zameer et al.).

Some of the articles in this special issue elucidate very effective, economical, eco-friendly and sustainable approaches to mitigate the abiotic stress in plants. Ahmad et al. demonstrated that bio-clay nanosheets infused with gibberellins mitigate the temperature and hexachlorobenzene stress in *Brassica alboglabra* plants. In an effort to promote drought tolerance in *M. oleifera* plants, Rehman et al. found that endophytic fungal consortia capable of synthesizing ACC deaminase improve stress tolerance through reducing the biosynthesis of ethylene and H_2_O_2_. Likewise, Ali et al. observed that endophytic fungus (*Stemphylium lycopersici*) enhances salinity tolerance stress tolerance in maize through regulating the metabolic and ionic status. Khan et al. examined the role of various soil amendments with bentonite clay to diminish uptake of heavy metal contaminants by *Solanum melongena* growing in tannery polluted conditions. The exogenous application of some antioxidants may alleviate abiotic stress in crop plants. The exogenous application of proline enhances yield of maize crop by reducing the level of stress related biomarkers and improving morph-physiological responses under various irrigation systems (Ibrahim et al.). Foliar application of silica enhances drought stress tolerance in various *Oryza sativa* cultivars (El-Okkiah et al.). Ali et al. described the role of phyto-melatonin in reduction of the heavy metal induced phytotoxicity. Correspondingly, exogenously applied silicon improves yield and water use efficiency of maize plants by alleviating salt stress (Alayafi et al.). Application of hydrogen sulfide assists banana fruits to tolerate chilling injuries by adjusting their ascorbate–glutathione cycle and γ-aminobutyric acid shunt pathway (Ali et al.). Hammad et al. evaluated the role of nitrogen on physiochemical activities and growth of maize plants subjected to abiotic stress. While, Gillani et al. demonstrated the brassinosteroids induced drought stress tolerance in maize through modulation of the expression level of drought-responsive genes. Furthermore, foliar application of folic acid improves biochemical activities, growth and oil content of *Coriandrum sativum* subjected to drought stress (Khan et al.). Khilji et al. also reported the stress ameliorative potential of fulvic acid through declining chromium, cadmium, and lead uptake in *Brassica napus* growing in paper sludge contaminated regimes.

The articles included in this special issue unveil that tolerant plants to alleviate stresses through accumulating toxins within their root zone, reducing translocation of toxins in upper parts, and by modulating their physiochemical as well as metabolic activities besides changing gene expression levels to decline or manage environmental stresses. Though, appropriate management of environmental stresses in food crops so far remains one of the great challenges throughout the world. Sooner or later, the foremost research may be focused to further recognize and illustrate the role of omics, transcriptomics and genomics in development of abiotic stress tolerant varieties having capability to reduce the uptake and translocation of phytotoxicants especially in their edible parts. Altogether, this special issue includes a set of virtuous research work, inspiring the scientific information about the environmental extremes and food crop production. Now, this Special Issue contains a set of very fascinating articles that uplift the information on the matter, and which would be beneficial for the society as well as for researchers.

## Author contributions

NAY and RS have made a substantial contribution to the concept or design of the article. MA and RS drafted the article. TAK and AA revised it critically for important intellectual content. All authors read and approved the final manuscript.
